# Prenatal diagnosis of achondrogenesis type I: a case report

**DOI:** 10.1186/1757-1626-1-406

**Published:** 2008-12-18

**Authors:** M Zeki Taner, Mertihan Kurdoglu, Cagatay Taskiran, M Anil Onan, Guven Gunaydin, Ozdemir Himmetoglu

**Affiliations:** 1 Department of Obstetrics and Gynecology, Gazi University School of Medicine, Ankara, Turkey; 2 Department of Obstetrics and Gynecology, Yuzuncu Yil University School of Medicine, Van, Turkey (Formerly, Department of Obstetrics and Gynecology, Gazi University School of Medicine, Ankara, Turkey); 3 Department of Obstetrics and Gynecology, Tunceli State Hospital, Tunceli, Turkey (Formerly, Department of Obstetrics and Gynecology, Gazi University School of Medicine, Ankara, Turkey)

## Abstract

**Introduction:**

Achondrogenesis is a lethal osteochondrodysplasia characterized by hypoplasia of the bones and is associated with various anomalies varying in severity. Based on clinical, radiologic, and histopathologic features, two types are distinguished.

**Case presentation:**

The prenatal ultrasound examination of a 32-year-old Turkish woman who was referred to our clinic at 33 weeks and 6 days of gestation revealed fetal micromelia together with several other anomalies. The female baby died shortly after birth and was diagnosed with achondrogenesis type I based on the clinical and radiographic findings.

**Conclusion:**

Ultrasonography is important in prenatal diagnosis and for distinguishing lethal skeletal dysplasias in order to counsel the parents about future recurrent risks. As it is a uniformly lethal disease, a definitive prenatal diagnosis of achondrogenesis may be an indication for pregnancy termination.

## Introduction

Achondrogenesis is a skeletal dysplasia, characterized by extremely shortened limbs, a normal to poorly ossified skull, a poorly ossified spine and pelvis, and severe pulmonary hypoplasia [1]. The incidence of achondrogenesis is 1 in 40 000 live births [2]. Although types I and II have been distinguished based on clinical, radiologic, and histopathologic features, there is considerable phenotypic heterogeneity in this disorder [3]. We report a case of the prenatal diagnosis of type I achondrogenesis (Parenti-Fraccaro) in a 33-week and 6-day-old fetus.

## Case presentation

A 32-year-old Turkish woman, gravida 3, para 2, was referred to our prenatal diagnosis and treatment clinic because of the diagnoses of gestational diabetes mellitus and polyhydramnios, and abnormal results of an ultrasound examination performed by her obstetrician. First-degree consanguinity was noted between the couple. Her first pregnancy had ended in a cesarean section at 33 weeks' gestation with a diagnosis of severe preeclampsia and breech presentation. Although it had not been possible to make an exact diagnosis, the appearance of the male baby had suggested dwarfism. The baby had died within 3 hours of birth. Her second pregnancy had been complicated by polyhydramnios, preeclampsia, and gestational diabetes mellitus and had ended in the birth of a healthy male baby.

**Figure 1:**
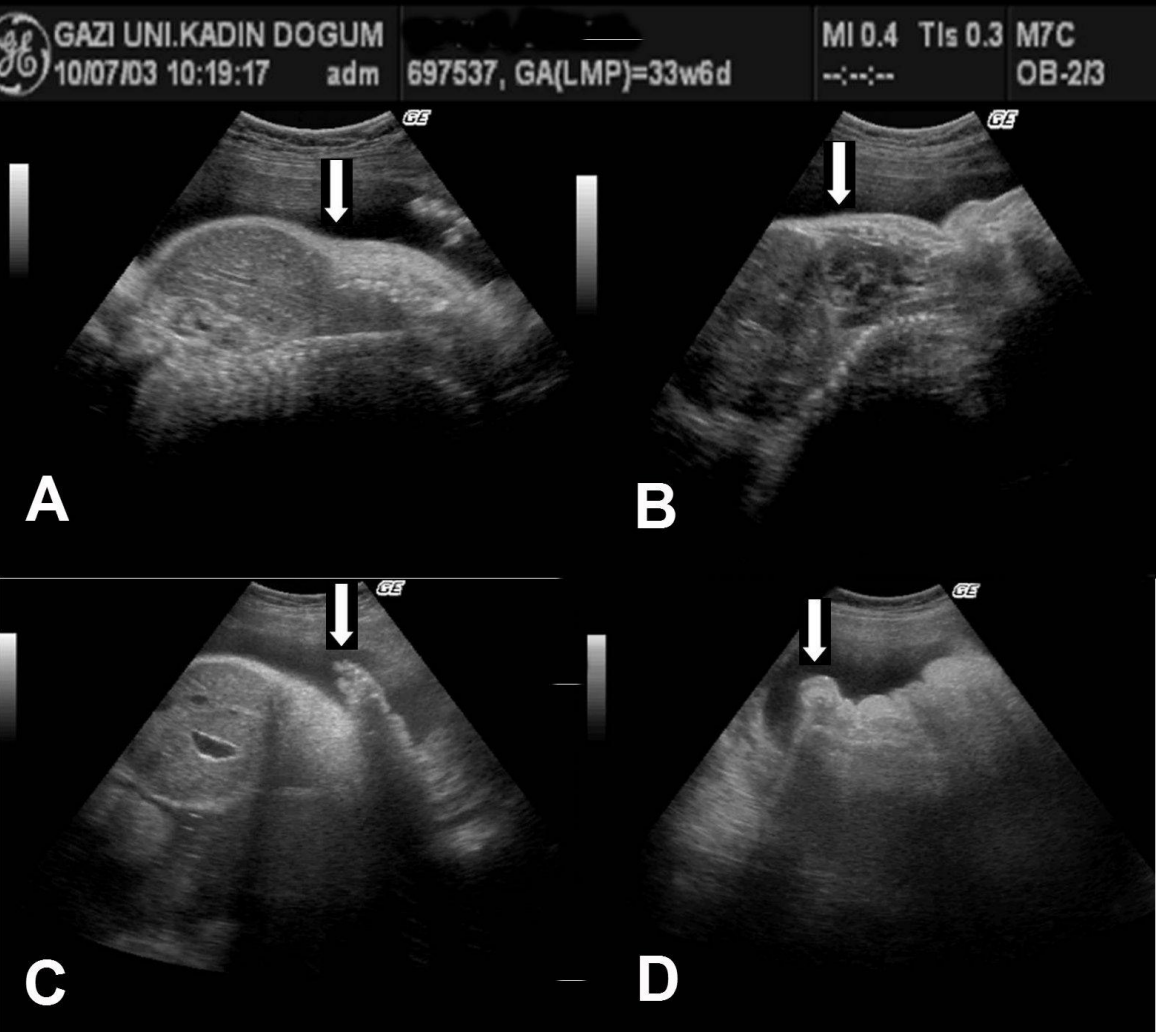
High abdomen/thorax ratio (A), pulmonary hypoplasia and narrow thorax (B), extreme micromelia (C, D) are observed in the prenatal sonogram at 33 weeks and 6 days of gestation.

**Figure 2:**
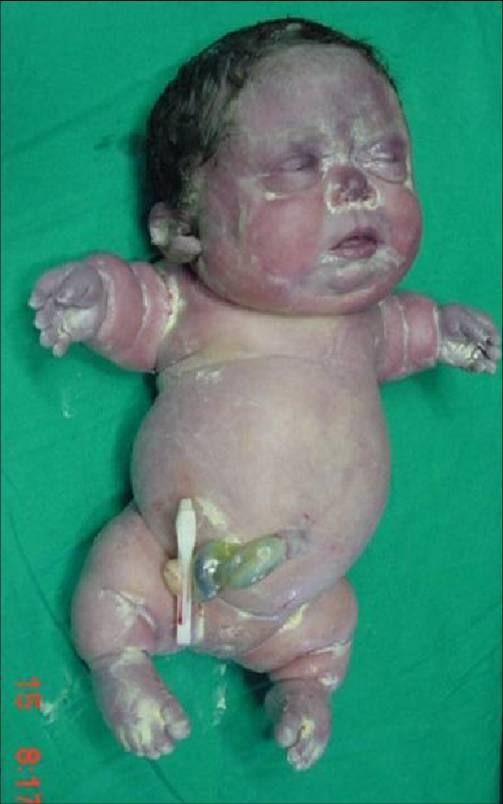
The appearance of the baby after birth.

**Figure 3:**
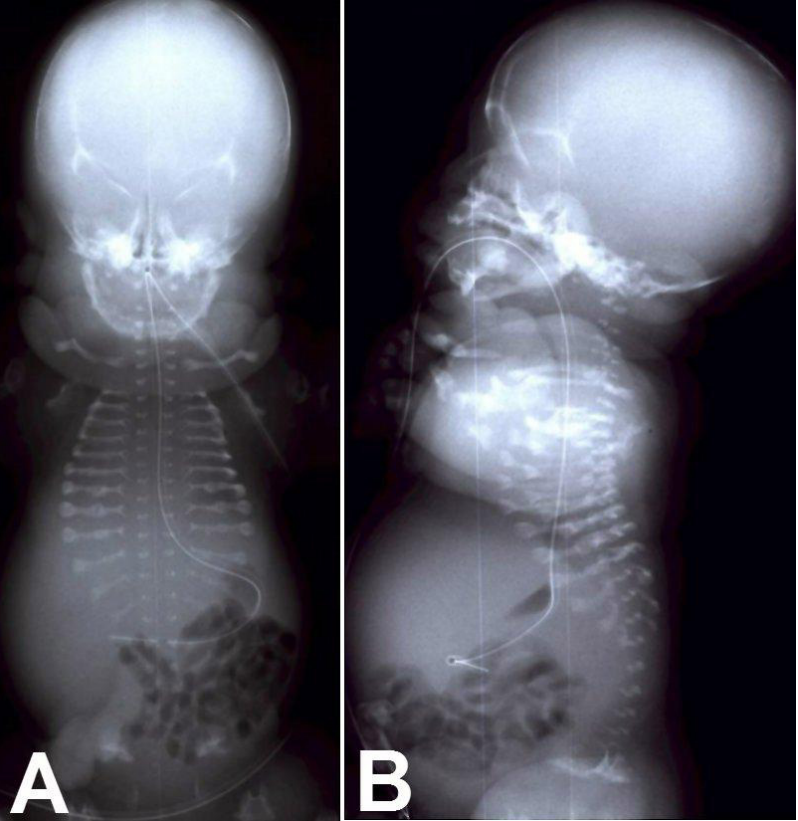
Postmortem anteroposterior (A) and lateral (B) whole-body radiographs of the baby.

Prenatal sonograms of the fetus of the present gestation revealed extreme micromelia, narrow thorax, high abdomen/thorax ratio, pulmonary hypoplasia, and poor mineralization of the skull and vertebrae. Polyhydramnios and a pseudo-hydropic appearance, pleural and pericardial effusion, and low lying ears were also observed (Figure 1). During the evaluation of the patient with a presumptive diagnosis of achondrogenesis, spontaneous labor started and she gave birth to 1810 gram and 31 centimeter female baby at 34 weeks and 4 days delivered by cesarean section. The baby died within the first thirty minutes of birth. Examination of the baby demonstrated hydrops, severe tetramicromelia, and a flat face. The head was disproportionately large relative to the reduced neck, trunk and limb length (Figure 2). Anteroposterior and lateral postmortem whole-body radiographs (Figure 3) revealed inadequate ossification of the bones, except for the clavicles, and there were short beaded ribs with multiple fractures, minimal ossification of vertebral bodies, arched iliac wings, stellate femurs and humeri, and micromelic long bones. All of these findings confirmed the prenatal sonographic findings.

## Discussion

Chondrodysplasias affect the growing cartilage, with an incidence of 1 to 3 in 10,000 births, and form a heterogeneous group of genetic origin skeletal dysplasias. Most chondrodysplasias are caused by mutations in various types of collagen genes. Collagen type II is coded by a large gene COL2A1 on 12q13.11-q13.12 and defects in collagen type II are caused by point mutations of this gene. The pathological changes in patients are observed in the growth plate, nucleus pulposus, and vitreous body, where the abnormal collagen type II is distributed [4].

Achondrogenesis, a lethal form of congenital chondrodystrophy, is characterized by extreme micromelia. The prenatal diagnosis of achondrogenesis is based on additional findings of narrow thorax, and poor mineralization of the skull and vertebrae. Polyhydramnios and a pseudo-hydropic appearance are also common [1]. These features are consistent with our findings.

Achondrogenesis may be differentiated from other skeletal dysplasias by having the most severe degree of limb shortening. The demineralization is only a differential diagnosis in osteogenesis imperfecta and hypophosphatasia, which do not present with the same degree of limb shortening [1].

With a considerable phenotypic heterogeneity seen in achondrogenesis [3], types I and II are distinguished based on clinical, radiologic, and histopathologic features. Achondrogenesis type I (Parenti-Fraccaro) is inherited autosomal recessive and is the more severe form, characterized by inadequate ossification of the skull, spine, and pelvis, extensive shortening of tubular bones, and multiple rib fractures. Achondrogenesis type II (Langer-Saldino) is characterized by various degrees of calcification of the pelvis, skull, and spine without rib fractures, and most type II cases are sporadic (new autosomal dominant mutations) [1].

Characterization of demineralization is important for differentiating between type I and II cases. When the demineralization affects the skull and iliac wings the presumptive diagnosis is type I; when the skull appears normally mineralized the presumptive diagnosis is type II. When demineralization is present on sonography, an X-ray may confirm it. However, in the absence of demineralization on ultrasound, radiological demineralization cannot be presumed. Since the recognition of demineralization by ultrasound is fraught with false negatives, there will be a tendency to over-report the type II form [1]. In our case, demineralization of the skull and iliac wings and multiple rib fractures observed in both ultrasonographic and radiological images support the diagnosis of type I achondrogenesis. Consanguinity of the parents also confirms our diagnosis since type I is autosomal recessively inherited.

Many cases of fetal skeletal dysplasias detected antenatally with ultrasonography have been reported. A final diagnosis was obtained in most cases by fetopathological examination and radiographic studies and molecular testing as deemed necessary. Also well recognized antenatally in this series was achondrogenesis with type II predominance. Witters et al. have described 38 dysplasias of which two cases were achondrogenesis type II, diagnosed at 12 and 18 weeks [5]. In the series of Puri et al. evaluating 15 cases with short limbed dwarfism, there were two cases of achondrogenesis syndromes, type Ia and type II-hypochondrogenesis overlap which were detected at 14 and 19 weeks, respectively [6].

Similar to the other lethal short limb dysplasia, achondrogenesis is lethal due to pulmonary hypoplasia [1]. Infants are either stillborn or die in the neonatal period, like in our case. Therefore, the pregnancy can be managed as other pregnancies with fatal outcome [1] and the option of pregnancy termination may be offered at any time after a definitive diagnosis.

## Conclusion

Specific prenatal diagnosis by ultrasound is helpful in distinguishing lethal skeletal dysplasias for counseling parents about future recurrent risks.

## Consent

Written informed consent was obtained from the patient for publication of this case report and accompanying images. A copy of the written consent is available for review by the Editor-in-Chief of this journal.

## Competing interests

The authors declare that they have no competing interests.

## Authors' contributions

MZT diagnosed and managed the case with full responsibility, and analyzed and interpreted the patient data. MK assisted MZT in the management of the case and was the major contributor in writing the manuscript. CT contributed to the writing and editing of the manuscript. GG assisted MZT in the management of the case and provided data and accompanying images. OH was the general consultant and advised in the management of the case and writing of the manuscript. All the authors read and approved the final manuscript.
